# Response to Savel’s “Reconsidering the Scope and Structural Integration of Leadership Education for Clinicians”

**DOI:** 10.5041/RMMJ.10582

**Published:** 2026-07-31

**Authors:** Nabilah Maher Chowdhury

**Affiliations:** Medical Education Department, Barking, Havering and Redbridge University Hospitals NHS Trust, London, United Kingdom

**Keywords:** Education, leadership, physician

**To the Editor**,

I read with great interest the narrative review by Savel (2025)^1^ examining formal education in leadership and management for practicing clinicians, particularly those working in critical care settings. The article provides a helpful overview of leadership styles, management principles, financial literacy, and strategic thinking, while also outlining formal educational pathways such as MBA programs and leadership certificate courses. Overall, the review offers practical guidance for clinicians who may be considering a more structured pathway into leadership roles within healthcare.

While the review clearly describes the educational opportunities available, several broader conceptual issues may merit further discussion.

First, the article presents leadership education largely as a voluntary pursuit, often undertaken later in a clinician’s career as administrative responsibilities emerge, typically through additional qualifications. However, a growing body of literature suggests that leadership should be considered a core clinical competency rather than an optional skill. For example, competency frameworks in medical education increasingly recognize leadership as an essential part of professional development.^2^ Studies in graduate medical education have also shown that structured leadership training incorporated into residency programs can improve team functioning, communication, and patient safety culture.^3^ Framing leadership education primarily as a mid-career activity may therefore risk reinforcing the idea that administrative competence is separate from, rather than being integrated into, clinical professionalism.

Second, while the review helpfully distinguishes between leadership and management, research suggests that these roles often overlap in practice. Clinicians who take on administrative responsibilities frequently move between strategic leadership and operational management as part of their everyday work.^4^ Similarly, Ackerly et al. emphasize the importance of blended competencies rather than treating leadership and management as entirely separate skill sets.^5^ In this context, competency-based development models that integrate both areas may better reflect the realities of modern healthcare leadership.

The review also discusses strategic frameworks such as Porter’s Five Forces.^6^ While these models can provide useful analytical tools, healthcare systems differ in important ways from traditional competitive industries. Healthcare organizations operate within complex regulatory environments and are guided by ethical responsibilities that extend beyond market competition. As Enthoven and others have noted, applying business strategy models directly to healthcare may therefore require careful adaptation to account for these structural and moral considerations.^7^

Savel’s review makes a valuable and timely contribution by bringing together a wide range of leadership and management concepts in an accessible and practical guide for clinicians. Building on this important foundation, future discussions may benefit from exploring how leadership training can be more fully integrated into medical education and how business frameworks can be adapted to the unique ethical and organizational realities of healthcare. By continuing to develop these ideas, the work initiated in this review has the potential to further strengthen the preparation of clinicians for the increasingly complex leadership roles they are asked to undertake.
